# A Reflection on Movement Disorders Fellowship Training in Deep Brain Stimulation: Past and Future

**DOI:** 10.1212/NE9.0000000000200299

**Published:** 2026-02-02

**Authors:** Deepal Shah-Zamora, Abhimanyu Mahajan, Harini Sarva

**Affiliations:** 1 Department of Neurology, Atrium Health Wake Forest Baptist, Winston-Salem, NC;; 2 Department of Neurology and Rehabilitation Medicine, University of Cincinnati, OH; and; 3 Department of Neurology, Weill Cornell Medicine, New York, NY.

## Abstract

Deep brain stimulation (DBS) has been an integral part of movement disorders care for decades. However, differences exist in techniques for surgical implantation of DBS and clinician experience with DBS systems, including use of new software, programming approaches, and postsurgical management of patients. DBS technologies have been rapidly advancing, and indications for DBS are increasing, including for psychiatric symptoms and epilepsy. The heterogeneity in the scope and utility of DBS is perhaps mirrored in education and training, despite efforts to develop competency measures for trainees. These advancements in DBS and the varying opportunities offered at each fellowship contribute to challenges for program directors to establish and implement consistent expectations. Similar challenges have been observed in other fields using neuromodulation. In this review, we seek to discuss the history and evolution of DBS therapy for movement disorders and in parallel, fellow training in DBS, particularly for movement disorders. The goal is to identify specific discrepancies in training that exist to ensure proficiency in the care of people with movement disorders and neuromodulation in general.

## Evolution of Deep Brain Stimulation

Deep brain stimulation (DBS) uses implanted electrodes to modulate current in targeted structures of the brain to address certain neurologic and psychiatric symptoms. Since reports of thalamic stimulation controlling tremor in the 1980s, DBS has been established as an efficacious treatment for medication-refractory Parkinson disease (PD) and dystonia.^1,2^ In the United States, the Food and Drug Administration (FDA) granted approval to Medtronic Inc. for thalamic DBS at the ventral intermediate nucleus in 1997 for treatment of essential tremor and PD-related tremor.^2^ In 2002, subthalamic nucleus and globus pallidus internus (GPi) targets were approved for treatment of PD.^2^ In 2003, the FDA granted a humanitarian device exception (HDE) for the use of GPi DBS for primary generalized and segmental dystonia.^2^ After 2016, Abbott Laboratories and Boston Scientific Corporation developed DBS systems with newer capabilities.^1,2^ The FDA also granted HDE approval of anterior limb of internal capsule DBS for obsessive compulsive disorder in 2009 and of anterior nucleus of thalamus DBS for refractory epilepsy in 2018.^2^

Surgical techniques for DBS implantation have evolved over time leading to a variety of practices. With advancements in neuroimaging including higher resolution MRI, presurgical planning transitioned from indirect (using standard anatomic landmarks) to direct targeting.^1,3^ Microelectrode recording (MER) with intraoperative stimulation involves temporarily implanting microelectrodes to use neurophysiologic guidance to localize the target and deliver test stimulation to assess therapeutic and side effect thresholds in DBS electrode placement.^4^ With intraoperative MRI and MRI-compatible devices allowing for real-time anatomic localization and adjustments during surgery, its utility has been debated.^3,5,6^ In a survey assessing real-world practices, only 15% of neurosurgeons in North America reported using intraoperative MRI.^7^ Fifty-five percent of the same survey participants reported that a movement disorders specialist was present during surgery and MER was used for electrode placement.^7^ Efforts to improve targeting by using tractography are ongoing.^3^ New and dual targets are being explored given development of DBS leads with increased number of contacts and variable contact spacing.^6^ In addition, dual-channel implantable pulse generators (IPGs) can control bilateral DBS leads, and all 3 major manufacturers offer rechargeable IPGs.^1^

Programming approach has also changed substantially. All 3 manufacturers have directional electrodes (segmented active contacts), which when used with upgraded software, allow fine-tuning of stimulation to improve efficacy and reduce stimulation-induced adverse effects.^6^ Abbott developed a decision-support software tool (Informity™) to aid in selection of the most favorable contact, and later, the option to perform virtual DBS adjustments.^6^ Medtronic developed sensing electrodes that record and live stream local field potentials for electrophysiology-based programming (BrainSense™).^6^ Visualization tools are available for image-guided programming with Medtronic (SureTune™) and Boston Scientific (GUIDE™ XT and Illumina 3D™).^6^ In 2025, the FDA approved commercial use of Medtronic's adaptive DBS, which allows for real-time dynamic stimulation adjustments based on an individual's PD symptoms and associated neuronal signals.^8^

To assess variability in practice due to these innovations, the Parkinson Study Group's (PSG) Functional Neurosurgical Workgroup surveyed DBS experts on programming patterns.^9^ Many had similar practices for treating fluctuations and dyskinesias with primary adjustments in amplitude. Meanwhile, there were differences between those with earlier fellowship training and recent graduates in use of newer technologies and troubleshooting for freezing of gait.^9^ Although patient care was not greatly affected, potential research opportunities may be concentrated in a few DBS centers depending on the utilization of specific technologies.

Given the many advancements in DBS since its inception, we seek to discuss the history and current state of movement disorders training in DBS. We aim to identify specific variabilities in training exposure and assessment, compare challenges faced in other specialties using DBS, and explore potential opportunities to ensure proficiency in comprehensive patient care.

## History and Current State of Movement Disorders DBS Training

“Movement disorders” as a field was named in the 1960s.^10^ The early pioneers of the field created educational opportunities in the 1980s including lectures at the American Academy of Neurology (AAN) annual meetings and sharing video recordings of people with unusual movements in small groups to discuss phenomenology.^10^ Larger-scale efforts included the founding of The International Parkinson and Movement Disorder Society (MDS) and its associated journals in the 1980s. In 1991, movement disorders leaders organized the first annual “Comprehensive Review of Movement Disorders for the Clinical Practitioner” in Aspen, Colorado.^10^ Formal training of neurologists to become movement disorders specialists also began in the 1980s and was based on the apprenticeship model.

**Figure 1:**
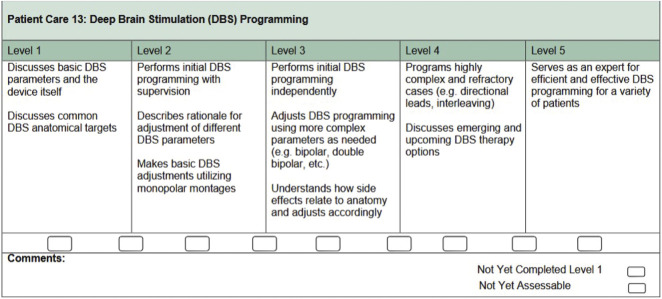
Competency Measures for Deep Brain Stimulation Training in Fellowship This figure describes the levels of competency for movement disorders fellows pertaining to their deep brain stimulation knowledge. It was originally published in 2022 as part of a supplement in “Viewpoint on Milestones for Fellowship Training in Movement Disorders” by Ratliff, et al. The figure was reprinted with permission from the authors. ©2022 The Authors. *Movement Disorders* published by Wiley Periodicals LLC on behalf of International Parkinson and Movement Disorder Society.

In North America, the apprenticeship-based model has since persisted.^11^ Movement disorders specialists seek funding from their institution and often from industry grants or philanthropy to train fellows.^11^ Currently, fellowship programs are 1 to 3 years long with varied expectations for learning relevant surgical approaches.^11^ A 2013 survey of North American movement disorders fellowships demonstrated variability in DBS training: 82.2% reported dedicated clinics for trainees to learn DBS programming, 62.2% offered intraoperative training, and about 28% of programs included a multidisciplinary DBS conference.^11^ To improve standardization of fellowship curriculum and milestones, the AAN developed the first core curriculum for movement disorders training in 2000.^12^ It did not include knowledge of surgical treatments such as DBS. In 2020, the AAN Movement Disorders Section and the MDS Pan-American Section collaborated to update the curriculum.^13^ The revised curriculum included interrogation of the DBS device, assessment of thresholds, and understanding target selection.^13^ Fellows were expected to screen for, recognize, and manage complications of DBS hardware, and stimulation-induced side effects. Learning intraoperative testing with MER was optional but encouraged. In May 2022, leaders in movement disorders training developed more specific fellow competency measures.^13^ Feedback from international movement disorders educators, primarily from the MDS, and North American fellowship directors was included in the updated 2022 milestones, which were aligned with previously published neurology core competency measures.^13^ (Figure 1) Despite the availability of clearly outlined competencies, their actual implementation by fellowship programs is unknown. Movement disorders fellowships are not formally accredited, which allows flexibility in training experiences while creating challenges in establishing and enforcing educational milestones. Institutional graduate medical education departments typically oversee nonaccredited programs. Furthermore, there are no written or oral board examinations in movement disorders to ultimately assess these competencies.

In addition to fellowship training, there are supplementary educational opportunities. These may also be of interest to residents seeking to learn DBS without further specialization. The MDS regional sections offer neuromodulation courses and online modules about DBS.^14^ The AAN annual meeting offers skilled teaching sessions on patient selection and programming. In addition, DBS device manufacturers offer courses specifically for movement disorders fellows. Topics may include DBS lead placement, cadaver laboratory simulation led by a neurosurgeon, and discussion of electrophysiology-based programming.^15^ The Greenville Neuromodulation Center offers an intensive course on intraoperative neurophysiologic monitoring for those seeking additional training in MER.^16^ These opportunities can cost hundreds to thousands of US dollars and have limited scholarship opportunities. Thus, cost and time commitments may not be feasible for everyone. Typically, there are no learning outcome assessments on course completion so their success in improving DBS knowledge is unknown.

## Future Directions and Potential Challenges

**Figure 2:**
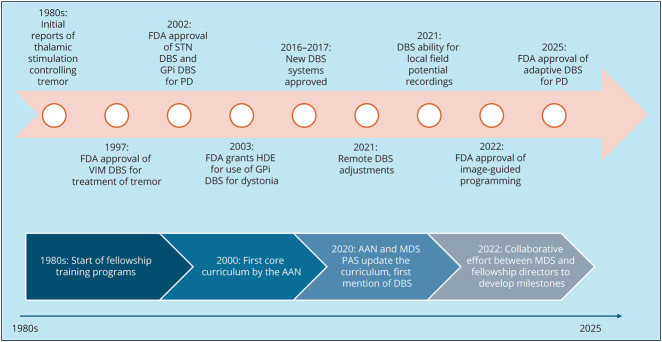
Evolution of Deep Brain Stimulation Technology and Movement Disorders Training This figure compares the major milestones within the field of deep brain stimulation (DBS) for movement disorders at the top with those for developing standardized, consistent DBS training for movement disorders fellows at the bottom. AAN = American Academy of Neurology; DBS = deep brain stimulation; FDA = Food and Drug Administration; GPi = globus pallidus internus; HDE = humanitarian device exception; MDS = The International Parkinson and Movement Disorder Society; PD = Parkinson disease; STN = subthalamic nucleus; VIM = ventral intermediate nucleus of thalamus.

The rapidly evolving advances in DBS technology and varied experiences offered at each fellowship create challenges for program directors to establish expectations (Figure 2). Nearly half of movement disorders fellowships in North America offer 1 year of training, limiting ability to spend time training in DBS.^11^ Variability in practices, including presence of a neurologist in the operating room, associated financial considerations, and availability of different DBS systems add to the conundrum for fellowship program directors. As such, standardization and regulation of competency metrics remain challenging.

Similar issues exist in accredited training programs as well. A survey of epilepsy fellowship directors noted variability in surgical procedure exposure, and most used faculty assessments instead of objective measures of fellow competency in epilepsy surgical procedures.^17,18^ In psychiatry, there is a growing interest in neuromodulation, yet current requirements only expect residents to understand the indications for neuromodulation and do not measure competency of performing the procedure or management afterward.^19,20^

All specialties using DBS would benefit from consistent and updated expectations of knowledge in DBS and neuromodulation. By graduation, trainees in neuromodulation should be competent in identifying appropriate surgical candidates, including target selection and potential associated adverse effects including evaluating postoperative wounds.^12,13^ This allows for better counseling of patients considering surgical intervention from preoperative assessments to postoperative evaluations. They should be able to differentiate between and operate various DBS manufacturers and become familiar with advancements in technology (such as the utility and limitations of adaptive DBS), as available for their specialty. Knowing the pros and cons of each device and how that can affect clinical outcomes is vital to helping patients decide which device is best for them. DBS trainees should gain exposure to various initial programming technologies, which can potentially increase clinical efficiency.^6^ Finally, they should be competent in initiating DBS therapy, performing initial troubleshooting, and be able to identify long-term hardware complications.^12,13^ This allows for better management of the whole disease process including concurrent medication adjustment. DBS trainees should also have an opportunity to familiarize themselves with intraoperative imaging and MER as it pertains to their specialty, so they can at least have more comprehensive discussions about DBS when practicing independently. These competencies can be assessed through ensuring a minimum number of cases per month, direct observation by faculty during clinical encounters, and review of milestone achievement during formal meetings between mentor and trainee.

The Brain Stimulation Subspeciality Summits convened in 2023 and 2024 and invited expert neurologists, psychiatrists, and neurosurgeons involved in translational or clinical research.^21^ From their meetings, they proposed creating a separate brain stimulation fellowship including training in DBS, transcranial magnetic stimulation, electroconvulsive therapy, vagal nerve stimulation, among others. Similar programs exist currently. The University of Toronto offers an Advanced Therapies of Movement Disorders clinical fellowship devoted to interventional therapies including DBS.^22^ Those who wish to pursue a more comprehensive fellowship in movement disorders receive 6 months of advanced therapies training. In psychiatry, there are dedicated fellowships in interventional psychiatry and neuromodulation.^23^ Although not stated explicitly, these programs appear to complement training within the original specialty. However, those already with clinical experience may seek to apply to a dedicated fellowship to improve their skills in neuromodulation. Fellowship directors in early meetings with trainees would be advised to gauge interest in these advanced programs and assist those interested in applying.

Alternatively, a network of “DBS fellows” may be established across North America as program directors identify movement disorders fellows interested in advanced DBS training and research. Typically, fellows complete a 2-year fellowship if interested in DBS. In this focused fellowship year, local (North American) DBS experts could provide mentorship and help ensure adequate training to this dedicated cohort during MDS or PSG meetings. A virtual educational opportunity could provide networking and mentorship to ensure exposure to a breadth of DBS practices outside of their institution. During the COVID-19 pandemic, a similar online educational series for all movement disorders fellows in the US was feasible and well received.^24^ This model could also be adopted in psychiatry and epilepsy and helps bridge a geographic educational gap in regions of the country with few DBS experts.

In the United States, there has been a consistent increase in DBS surgeries, with over 5,500 admissions per year.^25^ With the increasing prevalence of people with neurodegenerative conditions and expanding DBS indications, there is a clear need for neuromodulation in an aging population. Thus, it is necessary to increase interest and knowledge in DBS. Unfortunately, surveys of medical students' knowledge and perception of DBS demonstrate limited experiences.^26,27^ In a 2017 survey, of 127 neurology resident respondents, 37% reported no DBS exposure and only 2.4% felt confident about using DBS independently.^28^ In contrast, the expanding use of DBS, advancements in devices, surgical techniques and programming, and effect of DBS on patient quality of life highlight the growing need for DBS-trained clinicians.^29^ As neurology and psychiatry increasingly use neurosurgical interventions for chronic disease management, it is imperative that educators invest in a consistent and flexible approach to training in neuromodulation to ensure optimal, evidence-based care for our patients.

## Glossary

AAN: American Academy of Neurology
DBS: deep brain stimulation
FDA: Food and Drug Administration
GPi: globus pallidus internus
HDE: humanitarian device exception
IPG: implantable pulse generator
MDS: International Parkinson and Movement Disorder Society
MER: microelectrode recording
PD: Parkinson disease
PSG: Parkinson Study Group

## References

[1] Cavallieri F, Mulroy E, Moro E. The history of deep brain stimulation. Parkinsonism Relat Disord. 2024;121:105980. doi:10.1016/j.parkreldis.2023.105980 38161106

[2] Miocinovic S, Somayajula S, Chitnis S, Vitek JL. History, applications, and mechanisms of deep brain stimulation. JAMA Neurol. 2013;70(2):163-171. doi:10.1001/2013.jamaneurol.45 23407652

[3] Isler C, Bas G. New radiological techniques for planning of deep brain stimulation. Deep Brain Stimulation. 2024;4:24-28. doi:10.1016/j.jdbs.2023.12.004

[4] Fejeran J, Salazar F, Alvarez CM, Jahangiri FR. Deep brain stimulation and microelectrode recording for the treatment of parkinson's disease. Cureus. 2022;14(8):e27887. doi:10.7759/cureus.27887 36110462 PMC9464012

[5] Verhagen Metman L, Slavin KV, Rosenow JM, Vitek JL, van den Munckhof P. More than just the level of consciousness: comparing asleep and awake deep brain stimulation. Mov Disord. 2021;36(12):2763-2766. doi:10.1002/mds.28806 34585783

[6] Merola A, Singh J, Reeves K, et al. New frontiers for deep brain stimulation: directionality, sensing technologies, remote programming, robotic stereotactic assistance, asleep procedures, and connectomics. Front Neurol. 2021;12:694747. doi:10.3389/fneur.2021.694747 34367055 PMC8340024

[7] Howell S, Tabibian BE, Mooney JH, et al. Survey of practice preferences in deep brain stimulation surgery in the United States. Interdiscip Neurosurg. 2022;28:101463. doi:10.1016/j.inat.2021.101463

[8] Medtronic earns U.S. FDA Approval for the World's First Adaptive Deep Brain Stimulation System for People with Parkinson's. Medtronic News; 2025. Accessed April 17, 2025. news.medtronic.com/2025-02-24-Medtronic-earns-U-S-FDA-approval-for-the-worlds-first-Adaptive-deep-brain-stimulation-system-for-people-with-Parkinsons

[9] Cunningham JE, Cabrera LY, Mahajan A, et al. Survey of common deep brain stimulation programming practices by experts in Parkinson's disease. J Neurol. 2024;272(1):49. doi:10.1007/s00415-024-12751-0 39666124

[10] Fahn S. Interview with Doctor Stanley Fahn by Interviewed by Doctor Michael S. Okun and Lauren E. Klaffke, Interviewed for the American Academy of Neurology Oral History Project. 2015.

[11] Shih LC, Tarsy D, Okun MS. The current state and needs of North American movement disorders fellowship programs. Parkinsons Dis. 2013;2013:701426. doi:10.1155/2013/701426 23984186 PMC3745959

[12] American Academy of Neurology Movement Disorders Section. Movement Disorders Core Curriculum. AAN.com; 2000. Accessed June 21, 2024. aan.com/siteassets/home-page/tools-and-resources/academic-neurologist--researchers/teaching-materials/aan-core-curricula-for-program-directorstor/movement-dis-fellowship-core-curricula_tr.pdf

[13] Ratliff JB, Schaefer SM, Chitnis S, et al. Viewpoint on milestones for fellowship training in movement disorders. Mov Disord. 2022;37(8):1605-1609. doi:10.1002/mds.29146 35816077 PMC9543200

[14] International Parkinson and Movement Disorder Society (MDS). MDS-PAS 3rd School on Neuromodulation for Movement Disorders–Overview; 2022. movementdisorders.org. Accessed March 15, 2025. education.movementdisorders.org/Detail/590/MDS-PAS-3rd-School-on-Neuromodulation-for-Movement-Disorders

[15] Medtronic. Upcoming educational programs; 2025. Medtronic Academy. Accessed March 15, 2025. medtronicacademy.com/en-us/content/dbs-events/DBS

[16] Greenville Neuromodulation Center. Intensive Course on Intraoperative Neurophysiological Monitoring; 2025. Greenville Neuromodulation Center. Accessed March 15, 2025. greenvilleneuromodulationcenter.com/ionm-course/.

[17] Katyal R, Sheikh IS, Gutierrez C, et al. Epilepsy surgery education: a survey of US epilepsy fellowship program directors. J Clin Neurophysiol. 2026;43(1):1-8. doi:10.1097/WNP.0000000000001144 39934975

[18] Lemus HN, Dworetzky BA, Bubrick EJ, Cosgrove GR, Tobochnik S. Education research: evaluation of epilepsy surgery education in epilepsy and clinical neurophysiology fellowship programs. Neurol Educ. 2022;1(2):e200018. doi:10.1212/NE9.0000000000200018 40809504 PMC12339225

[19] Danilewitz M, Ainsworth NJ, Liu C, Vila-Rodriguez F. Towards competency-based medical education in neurostimulation. Acad Psychiatry. 2020;44(6):775-778. doi:10.1007/s40596-020-01195-z 32048176

[20] Egan D, Bailey KJ, Abu-Hamad S, et al. A model for formal residency training in interventional psychiatry. Acad Psychiatry. 2024;48(5):431-435. doi:10.1007/s40596-024-02058-7 39331231

[21] Siddiqi SH, Chen L, Trapp NT, et al. Towards accredited clinical training in brain stimulation: proceedings from the brain stimulation subspecialty summits. Brain Stimul. 2025;18(2):298-305. doi:10.1016/j.brs.2025.02.012 39988120 PMC13222746

[22] Advanced Therapies of Movement Disorders. University of Toronto Department of Medicine; 2021. Accessed November 14, 2025. deptmedicine.utoronto.ca/advanced-therapies-movement-disorders

[23] Department of Psychiatry & Behavioral Sciences. Neuromodulation Medicine Fellowship. University of Minnesota Education & Training; 2019. Accessed November 14, 2025. med.umn.edu/psychiatry/education-training/fellowships/neuromodulation-medicine

[24] Mahajan A, El-Nazer R, Chitnis S. An online education method for movement disorders during COVID-19: opportunity and experience. Mov Disord. 2021;36(7):1475-1480. doi:10.1002/mds.28648 33938588 PMC8242782

[25] Sarica C, Conner CR, Yamamoto K, et al. Trends and disparities in deep brain stimulation utilization in the United States: a nationwide inpatient sample analysis from 1993 to 2017. Lancet Reg Health Am. 2023;26:100599. doi:10.1016/j.lana.2023.100599 37876670 PMC10593574

[26] Wloch A, Saryyeva A, Heissler HE, Schrader C, Capelle HH, Krauss JK. What do medical students know about deep brain stimulation? Stereotact Funct Neurosurg. 2017;95(2):125-132. doi:10.1159/000464254 28434004

[27] Saway BF, Monjazeb S, Godbe K, Anwyll T, Kablinger A, Witcher M. Medical students' knowledge and perception of deep brain stimulation. J Med Educ Curric. 2021;8:2382120521989977. doi:10.1177/2382120521989977 PMC7930653 33718611

[28] Mahajan A, Cahill C, Scharf E, et al. Neurology residency training in 2017: a survey of preparation, perspectives, and plans. Neurology. 2019;92(2):76-83. doi:10.1212/WNL.0000000000006739 30518554

[29] Jost ST, Aloui S, Evans J, et al. Neurostimulation for advanced parkinson disease and quality of life at 5 years: a nonrandomized controlled trial. JAMA Netw Open. 2024;7(1):e2352177. doi:10.1001/jamanetworkopen.2023.52177 38236600 PMC10797423

